# Trauma-related falls in an urban geriatric population: predictive risk factors for poorer clinical outcomes

**DOI:** 10.1186/s40621-023-00418-9

**Published:** 2023-01-30

**Authors:** Alexander Farrell, Taylor Castro, Shreya Nalubola, Nisha Lakhi

**Affiliations:** 1 Touro University College of Osteopathic Medicine, Middletown, NY USA; 2 College of Osteopathic Medicine, New York Institute of Technology, Old Westbury, NY USA; 3 School of Medicine, New York Medical College, Valhalla, NY USA; 4 Department of Trauma Surgery, Richmond University Medical Center, Staten Island, NY USA

**Keywords:** Elderly, Falls, Polypharmacy, Risk factors, Adverse outcome, Trauma

## Abstract

**Background:**

The aim of this study was to elucidate associations between polypharmacy, types of medications, and geriatric comorbidities to identify predictive risk factors for poorer clinical outcomes following trauma-related falls in the geriatric population. Nearly 80% of trauma-related hospital admissions in the older adult population are secondary to falls, accounting for 3 million emergency department visits annually. Numerous studies have demonstrated associations between falls, polypharmacy, and other geriatric comorbidities, but studies outlining predictive risk factors for poor clinical outcomes are lacking.

**Methods:**

A retrospective cohort study of 1087 patients ≥ 65 years old who presented to Level 1 Trauma Center after a trauma-related fall. Comorbidities, current medication, demographic information, and clinical outcomes were identified to ascertain predictive risk factors for poorer clinical outcomes. Variables were assessed for statistical significance on unadjusted analysis. Variables found to be significant were entered into a multivariable logistic regression model to test for adjusted associations, with *p* < 0.05 as statistically significant, and presented as adjusted odds ratios with 95% confidence intervals.

**Results:**

Polypharmacy ≥ 4 medications (aOR 2.38 (1.10–5.15), *p* < .028) was an independent predictor of hospital readmission within 30 days. Chronic kidney disease, male gender, and Asian race had an increased association with ICU admission. History of malignancy (aOR 3.65 (1.62–8.19), *p* < .002) and chronic kidney disease (aOR 2.56 (1.11–5.96), *p* < .027) were independent predictors of 30-day mortality.

**Conclusions:**

Polypharmacy, chronic renal disease, malignancy history, male gender, and Asian race had an increased association of adverse clinical outcomes after falls in the geriatric population. Critical evaluation of patients with these risk factors may be needed to mitigate risk in this population.

**Supplementary Information:**

The online version contains supplementary material available at 10.1186/s40621-023-00418-9.

## Background

Unintentional injury is the 8th most common cause of death in the USA in individuals 65 years old or greater and fall-related injuries contribute to a significant portion of these injuries (Kannus et al. 2005; Moreland et al. 2020). Nearly 80% of trauma-related hospital admissions in the USA in the geriatric population are injuries related to falls. These falls account for nearly 1 million hospitalizations and nearly 3 million emergency department visits annually (Moreland et al. 2020). Injuries from these falls are associated with substantial morbidity and mortality in the geriatric population, causing both fatal and non-fatal injuries (Ziere et al. 2006; Zia et al. 2015). Almost 33% of individuals 65 years of age and older and 50% of individuals over 80 will experience a fall annually, suggesting that injuries related to these falls are becoming a public health concern that must not go overlooked (Xue et al. 2021; Smith et al. 2017).

The pathophysiology of falls in the geriatric population is multifactorial, including both extrinsic causes (e.g., number of medications and their cumulative side effect profiles, home environment) and intrinsic causes (comorbidities, functional impairment, frailty) (Zia et al. 2015). The geriatric population is specifically susceptible to falls due to increased incidence of functional limitation secondary to musculoskeletal, neurological, and psychosocial factors (Ziere et al. 2006). A combination of all aforementioned factors contributes to the heavy prevalence of falls in this demographic (Ziere et al. 2006).

Numerous studies (Davies et al. 2020; Ziere et al. 2006; Zia et al. 2015; Xue et al. 2021) have demonstrated the association between falls and polypharmacy in the elderly population. Although there is substantial evidence for improved clinical outcomes from disease-specific medications, the cumulative side effect profile may contribute to the increased incidence of falls and, potentially, lead to increased morbidity and mortality associated with trauma injuries in the geriatric population (Moreland et al. 2020; Zia et al. 2015). Other research has indicated that certain comorbidities, including hypertension and diabetes mellitus, also serve as predictive risk factors for falls in older adults (Smith et al. 2017). However, detailed studies elucidating the associations between polypharmacy, types of medications, geriatric comorbidities, and adverse clinical outcomes are lacking. Thus, the primary objective of this study was to identify predictive risk factors for poorer clinical outcomes after falls in the geriatric population that presented to our trauma center.

## Materials and methods

This IRB-approved (Study # 14647) retrospective cohort study was conducted at Richmond University Medical Center, a Level 1 Trauma Center in Staten Island, New York, between the dates of March 2019 and March 2022. The protocol was reviewed and approved by the IRB of New York Medical College, Valhalla, New York. The primary objective of this study was to identify predictive risk factors for adverse clinical outcomes after falls in the geriatric population that presented to our Level 1 Trauma Center. The adverse outcomes examined were hospital admission, length of stay, necessity for intensive care unit admission, necessity for surgery, 30-day mortality, and readmission within 30 days for any health-related condition. None of our outcomes were treated as mutually exclusive, and all were categorized as independent events. Geriatric was defined as individuals aged 65 years and older. The medical records of all patients 65 years of age and older who presented to the trauma center with a primary diagnosis of a fall during the study period were identified using a trauma registry. Electronic medical record (EMR) review, which allowed access to all patient encounters, in conjunction with current procedural terminology (CPT) and International Classification of Diseases, 10th Revision, Clinical Modification (ICD-10-CM) codes, was used to identify demographic and comorbid factors included in the analysis.

Collected data points included age, gender, race, and/or ethnicity (as self-reported in EMR), body mass index (BMI), mechanism of fall (MOF), level of trauma activation, and number/type of medications prescribed at the time of presentation. Trauma activation is used within our Level 1 Trauma Center to categorize the severity of injury (Appendix 1). For the purposes of this study, polypharmacy was graded into two categories: ≥ 4 prescription medications and ≥ 10 prescription medications. Patients with polypharmacy ≥ 10 were included in both groups. The presence of comorbidities was identified using ICD-10-CM codes and included hypertension, hyperlipidemia, diabetes mellitus, heart disease (congestive heart failure (CHF), coronary artery disease (CAD), atrial fibrillation), chronic obstructive pulmonary disease (COPD), depression, chronic kidney disease/end-stage renal disease (CKD/ESRD), history of cerebrovascular accident (CVA), history of malignancy, and history of dementia. Medications analyzed were identified via EMR review and included current anticoagulant use (novel oral anticoagulants (NOAC), warfarin, and any formulary of heparin (unfractionated heparin, low molecular weight heparin)), antiplatelet use (ASA, other antiplatelet).

### Statistical analysis

Statistical analysis was carried out using IBM SPSS 28.0. Unadjusted analyses for continuous variables were compared using means. Bivariate variables were assessed via Student’s t tests. Categorical data were compared via chi-square, Mann–Whitney U test, and unadjusted logistic regressions. Associations between two continuous variables were assessed using Pearson’s correlation, to assess for collinearity. The goodness of fit was performed for all categorical variables using the chi-square test, with a *p* value of < 0.05 considered statistically significant. Variables that were statistically significant on unadjusted analysis were tested for the interaction of terms. Non-redundant variables were entered into a multivariable logistic regression model to test for adjusted associations (see Additional file 1: Tables S1–S5). This process was completed for each outcome assessed. Variables that retained statistical significance on multivariable regression were reported as adjusted odds ratios (aOR) with 95% confidence intervals.

### Power analysis

An a priori power analysis (“G*Power Erdfelder, Faul, * Buchner, Behavior Research Methods and Instruments & Computers, 1996) was performed using z test (logistic regression) for the statistical test. Based on data of Ziere et al. (2006) that demonstrated that polypharmacy increased the risk for falls, with an OR of 1.4 for 3 medications to 1.6 for 4 or more medications, we estimate a small–medium effect size of polypharmacy attributed to adverse outcomes. Assuming a two-tailed test with an 80% power and alpha = 0.05, the total number of 721 patients will be enough to provide a sample of sufficient power. We performed a post hoc power analysis to confirm the power of our study using polypharmacy as a risk for 30-day hospital readmission and found that the power of our study was 86.4%.

## Results

### Patient demographics and comorbidities

During the study period, 1087 patients aged ≥ 65 years presented to the trauma center for a trauma-related fall. Demographics are presented in Table 1. The mean age was 79.9 years (65.0–105.7 years) with a mean body mass index of 25.86 kg/m^2^ (13.60–55.10 kg/m^2^). The average number of medications prescribed at the time of presentation was 7.99 (0–39 medications). The class of medications prescribed, specific comorbidities, and mechanisms of falls are outlined in Table 1.

**Table 1:** Population demographics

Population demographic	Frequency	Percentage (%)
Gender		
Male	643	59.2
Female	443	40.8
Race		
White	772	71.9
Black	100	9.3
Asian	36	3.4
Native Hawaiian	2	0.2
Not specified	163	15.2
Ethnicity		
Non-Hispanic	946	86.9
Hispanic	141	13.1
Polypharmacy status		
Prescribed ≥ 4 medications	891	83.1
Prescribed ≥ 10 medications	366	34.1
Prescribed medication		
Antidepressant	181	16.9
Antihypertensive	737	68.6
Antiplatelet therapy (not ASA)	139	13
ASA	469	43.8
Diuretic	311	29
Heparin	18	1.7
Narcotic	76	7.1
NOAC	207	19.3
Warfarin	43	4
Comorbidity		
Anticoagulant therapy	368	34.3
Atrial fibrillation	216	20.1
Chronic obstructive pulmonary disease	157	14.6
Congestive heart failure	140	13
Coronary artery disease	308	28.7
Depression	186	17.3
Diabetes mellitus, type 2	371	34.5
End-stage renal disease/chronic kidney disease	167	15.6
History of cerebrovascular accident	130	12.1
History of dementia	296	27.5
History of malignancy	153	14.2
Hyperlipidemia	583	54.3
Hypertension	885	82.2
Outcome		
30-day mortality	27	2.5
Admitted again within 30 days	133	12.4
Admitted to inpatient service	873	80.8
Admitted to intensive care unit	145	13.4
Necessity for surgery	436	40.2
Mechanism of fall		
Unspecified	148	13.6
From same level	694	63.9
From stairs/step	94	8.7
From bed	81	7.5
From chair/wheelchair	45	4.1
From toilet	8	0.7
From ladder	5	0.5
Other	11	1.1
Trauma activation		
No activation	34	3.1
Level 1	15	1.4
Level 2	727	66.9
Level 3	310	28.5

Of the 1087 patients, 863 (80.8%) were admitted and had an average length of stay of 4.74 days (0–69 days). The rate of admission, necessity for intensive care unit admission, 30-day mortality, and readmission within 30 days are presented in Table 1.

### Inpatient admission

Of the 1087 patients in the study, 873 (80.8%) were admitted to an inpatient service. Age, race, and BMI of the patients had no significant effect on the rates of inpatient admission. There was a significant, positive association between inpatient admission and the number of medications prescribed, with a mean number of medications for patients admitted, 8.27 (0–39) versus not admitted, 6.86 (0–26), *p* < 0.001. On unadjusted analysis, polypharmacy ≥ 4 medications (82.6% vs. 71.1%, *p* < 0.001, OR 1.93, 95% CI 1.34–2.78), polypharmacy ≥ 10 medications (84.4% vs. 78.7%, *p* = 0.026, OR 1.46, 95% CI 1.04–2.04) and patients actively taking anticoagulation (85.1% vs. 78.3%, *p* = 0.008, OR 1.58, 95% CI 1.12–2.21) had a statistically significant increased risk of hospitalization. Conversely, heparin (61.1% vs. 81%, *p* = 0.034, OR 0.37, 95% CI 0.14–0.96), as well as antihypertensive use (78.5% vs. 85%, *p* = 0.013, OR 0.64, 95% CI 0.46–0.91) were significantly associated with decreased risk of admission. Two comorbidities found to be significantly associated with hospitalization included hyperlipidemia (84.4% vs. 76.1%, *p* < 0.001, OR 1.70, CI 1.25–2.29) and chronic kidney disease/end-stage renal disease (92.8% vs. 78.4%, *p* < 0.001, OR 3.55, 95% CI 1.93–6.57). Additional file 1: Table S1 presents the unadjusted analysis of factors associated with inpatient admission.

On adjusted analysis (Table 2), the following risk factors were found to retain statistical significance and are independent predictors of increased association of hospital admission: number of prescribed medications (aOR 1.08, 95% CI 1.01–1.16), hyperlipidemia (aOR 1.51, 95% CI 1.09–2.11), and ESRD/CKD (aOR 3.18, 95% CI 1.72–5.90). Both heparin (aOR 0.21, 95% CI 0.07–0.61) and antihypertensive use (aOR 0.42, 95% CI 0.28–0.62) retained statistical significance as factors associated with decreased risk of hospital admission.

**Table 2:** Multivariable regression analysis of risk factors

Outcome	Comorbidity/risk factor	Significance	Adjusted odds ratio with 95% CI
Risk of hospital admission	Number of medications	*p* < .038	1.08 (1.01–1.16)
	Heparin	*p* < .004	0.21 (0.07–0.61)
	Antihypertensive	*p* < .001	0.42 (0.28–0.62)
	Hyperlipidemia	*p* < .014	1.51 (1.09–2.10)
	ESRD/CKD	*p* < .001	3.18 (1.72–5.90)
Risk of intensive care unit admission	Male gender	*p* < .045	1.45 (1.01–2.08)
	Asian race	*p* < .018	1.79 (1.11–2.89)
	ESRD/CKD	*p* < .037	1.61 (1.03–2.51)
Risk of 30-day mortality	History of malignancy	*p* < .002	3.65 (1.62–8.19)
	ESRD/CKD	*p* < .027	2.575 (1.11–5.96)
Risk of readmission within next 30 days	Age	*p* < .007	0.97 (0.95–0.99)
	Polypharmacy ≥ 4	*p* < .028	2.38 (1.099–5.15)
	Antihypertensive use	*p* < .002	0.52 (0.35–0.79)
	Hyperlipidemia	*p* < .009	1.73 (1.15–2.60)

### Length of hospitalization

The average length of stay of patients admitted was 4.74 days (95% CI 4.37–5.11 days). Patient age, race, BMI, and the number of medications prescribed showed no significant impact on the length of hospitalization. However, male patients had a significantly longer hospital stay compared to females (5.32 days vs. 4.35 days, *p* = 0.012). Patients that were prescribed aspirin had a significantly shorter length of hospitalization compared to those not using aspirin (3.96 days (3.44–4.48) vs. 5.42 days (4.89–5.95)). However, patients receiving heparin prescription exhibited a significant increase in length of hospital stay (8.38 days (2.12–14.65) vs. 4.73 days (4.35–5.10)). No other medications studied exhibited significant alteration to the length of hospital stay. Both CKD/ESRD (6.84 days (5.74–7.94) vs. 4.36 days (3.97–4.75)) and COPD (6.21 days (4.76–7.65) vs. 4.52 days (4.16–4.88)) were significantly associated with increased duration of hospitalization. Additional file 1: Table S2 presents the unadjusted analysis of hospitalization lengths for risk factors.

On adjusted analysis (Table 2), all data points that were found to be statistically significant on unadjusted analysis retained statistical significance.

### Intensive care unit admission

Admission to the intensive care unit (ICU) was necessary for 145 (13.4%) patients. Male patients had a significantly increased association of ICU admission compared to females (16.3% vs. 11.4%, OR 1.51, 95% CI 1.04–2.15). Patients identifying as Asian also had a statistically significant higher rate of ICU admission compared to other ethnicities (*p* = 0.025). Age, BMI, and number of medications prescribed at the time of presentation exhibited no significant effect on ICU admission rates. Patients on warfarin were significantly more likely to need ICU level of care (25.6% vs. 13.0%, OR 2.31, 95% CI 1.14–4.69). With respect to comorbidities, patients with a previous diagnosis of atrial fibrillation (18.5% vs. 12.3%, OR 1.63, 95% CI 1.09–2.42), history of CVA, (19.4% vs. 12.7%, OR 1.65, 95% CI 1.02–2.66), or CKD/ESRD (19.9% vs. 12.4%, OR 1.75, 95% CI 1.14–2.67) had a significant risk of ICU admission (Additional file 1: Table S3). Patients admitted to the ICU had an increased 30-day mortality (11.0% vs. 1.2%, *p* < 0.001), but no difference in 30-day readmission compared to non-ICU admitted patients (15.2% vs. 12.0%, *p* = 0.279).

On multivariable/adjusted regression, male gender (aOR 1.45, 95% CI 1.01–2.08), Asian race (aOR 1.79, 95% CI 1.11–2.89) and a history of ESRD/CKD (aOR 1.61, 95% CI 1.03–2.51) retained statistical significance, indicating an independent prediction of ICU admission (Table 2).

### 30-day mortality

Of the 1087 patients, 27 (2.5%) mortalities were reported within 30 days of initial presentation. No demographic factors including gender, age, race, BMI, number of medications prescribed, or medication class affected 30-day mortality. However, patients with a known CAD (4.2% vs. 1.8%, OR 2.37, 95% CI 1.09–5.09), history of malignancy (6.5% vs. 1.8%, OR 3.72, 95% CI 1.67–8.28), or history of CKD/ESRD (5.4% vs. 2.0%, OR 2.81, 95% CI 1.24–6.37) had statistically increased association of 30-day mortality compared to those without. Additional file 1: Table S4 exhibits unadjusted risk of 30-day mortality for all risk factors analyzed.

History of malignancy (aOR 3.65, 95% CI 1.62– 8.19) and ESRD/CKD (aOR 2.56, 95% CI 1.11–5.96) retained statistical significance on adjusted regression and are independent predictors of 30-day mortality (Table 2).

### Readmission within 30 days

Readmission within 30 days of the initial presentation was required for 133 (12.4%) patients. Unadjusted analysis for predictors of readmission revealed that male gender (15.8% vs. 10.0%, OR 1.68, 95% CI 1.17–2.42), polypharmacy ≥ 4 medications (13.7% vs. 6.1%, OR 2.45, 95% CI 1.29–4.65), and polypharmacy ≥ 10 medications (16.7% vs. 10.2%, OR 1.76, 95% CI 1.22–2.54) had significantly increased rates of 30-day readmission. Aspirin use was the only prescribed medication that had an increased association of readmission within 30 days (14.7% vs. 10.6%, OR 1.45, 95% CI 1.01–2.09). With respect to comorbidities, hyperlipidemia (15.4% vs. 8.8%, OR 1.90, 95% CI 1.29–2.80) and congestive heart failure (CHF) (18.6% vs. 11.5%, OR 1.76, 95% CI 1.10–2.82) and CKD/ESRD (18.0% vs. 11.4%, OR 1.71, 95% CI 1.09–2.66) had a statistically significant increased association of readmission 30-day readmission. (Additional file 1: Table S5).

On adjusted analysis, polypharmacy ≥ 4 medications prescribed (aOR 2.38, 95% CI 1.10–5.15), history of hyperlipidemia (aOR 1.72, 95% CI 1.15–2.60), use of antihypertensive medications (aOR 0.52, 95% CI 0.34–0.79), were independent predictors of 30-day readmission (Table 2).

## Discussion

A principal objective of this study was to examine the role of polypharmacy in poorer hospital outcomes after a trauma-related fall. Unfortunately, polypharmacy remains a vague term with nearly 140 different definitions found throughout the scientific literature (Masnoon et al. 2017). We made the decision to qualify polypharmacy as ≥ 4 medications prescribed in a given period of time in concordance with numerous previous studies such as Ziere et al. (2006). As a way of assessing severity, we then decided to separate categories into ≥ 4 medications prescribed and ≥ 10 medications prescribed. Numerous studies have researched and exhibited the strong connection between polypharmacy and the rates of falls in the elderly population (Ziere et al. 2006; Zia et al. 2015). Our analyses revealed that polypharmacy is a significant risk factor for needing inpatient admission after a trauma-related fall, as well as an independent risk factor for readmission within 30 days. These results are in concordance with previous studies that have examined polypharmacy and demonstrated associations with several adverse clinical outcomes including increased falls, length of hospital stay, and readmission rates (Ziere et al. 2006; Zia et al. 2015; Milton et al. 2008; Freeland et al. 2012; Chang et al. 2020). Although our study did not find that the number of medications or polypharmacy increased overall mortality as other studies have (Campbell et al. 2004; Frazier 2005; Chang et al. 2020), we postulate that these effects were not realized secondary to the relatively short follow-up time of 30 days that was used in our study. On adjusted analysis, the number of medications prescribed significantly correlated with an increasing risk of hospitalization, with an increase in aOR of 1.1 per medication prescribed. These results are relatively consistent with previous studies that investigated fall risk with increasing medication burden. These studies displayed an 18% increase in falling per medication prescribed (Centers for Disease Control and Prevention 2022) and a 14% increase in risk per medication over 4 (Freeland et al. 2012), respectively. These findings only hint at the growing need for more research on the risks of poorer hospital outcomes secondary to heavy medication burden. We postulate that these poorer outcomes could be secondary to drug–drug interaction, drug–disease interaction, or perhaps comorbidities affecting functional status, leading to a poorer health status at baseline.

Furthermore, we investigated certain demographic factors that may predispose this population to poorer outcomes. Certain studies have elucidated the link between the male gender, along with older age, as a risk factor for severe injuries from ground-level falls (Kim et al. 2021). Our study expands upon these findings to show the significance of the male gender as a risk factor for poorer clinical outcomes after these falls. Specifically, our analyses revealed that the male gender is a significant risk factor for longer length of hospital stay, ICU admission, as well as readmission within 30 days. We additionally found that the Asian race is a significant risk factor for needing ICU admission after a trauma-related fall; however, 30-day mortality was not increased. We postulate this could be secondary to the small number of Asian patients in our study. Previous studies have only revealed white race to be a significant risk factor for falls (Fuller 2000) with outcomes-based studies related to race not reported.

In addition to polypharmacy and demographic factors, we found that certain medication classes were associated with adverse clinical outcomes. Leiss et al. (2015) researched the link between polypharmacy with concurrent anticoagulant use and found a statistically significant increased association of clinically relevant bleeding events in patients taking warfarin. Our results concur and expand upon these results as on unadjusted analysis warfarin use was found to be a risk factor for ICU admission after a fall. Charlton et al. (2018) similarly investigated the role of anticoagulants and clinical outcomes and found that warfarin use significantly increases hospitalization length by 2.0–2.6 days compared to NOACs. A 2022 meta-analysis (Carnicelli et al. 2022) established that NOACs are associated with a significantly lower risk of stroke, systemic embolism, intracranial bleeding, and death as compared to warfarin. Further prospective research on the use of NOACs compared to warfarin with respect to outcomes post-trauma-related fall may further clarify risks versus benefits.

Overall, our analyses revealed conflicting results regarding the connection between anticoagulant use and poorer clinical outcomes. Specifically, heparin was found to decrease the risk of hospital admission, but heparin use was also associated with an increased length of hospital stay on both unadjusted and adjusted analyses. We anticipate these conflicting results are secondary to a type I error due to the relatively small number of individuals prescribed heparin in this population (*n* = 18) as it is rarely used in an outpatient setting. Additionally, we found no significant association between anticoagulant use and 30-day mortality. Previous research including Jaspers Focks et al. (2016) has found that an increasing number of medications prescribed with concurrent anticoagulant use, increased all-cause mortality, independent of anticoagulant type (Charlton et al. 2018). We postulate these discrepancies may be due to the need for longer follow-up and larger population size.

Satisfactory blood pressure control has been shown to decrease rates of adverse cardiovascular and renal outcomes including stroke, chronic kidney disease, heart failure, and left ventricular hypertrophy (Flack and Adekola 2020; Izzo 2011). Our results demonstrate that patients on antihypertensive medications had a decreased risk of inpatient admission after a fall, as well as decreased risk of readmission within 30 days. It is unclear whether tighter blood pressure control contributed to the improved clinical outcomes as blood pressure was not an endpoint of this study. Although the previous literature (Bromfield et al. 2017) has shown that neither the number of antihypertensive medications nor baseline systolic or diastolic blood pressure was found to have a risk of serious falls in the geriatric population, antihypertensive medication use is approached with caution in the older adult population secondary to possible dysautonomia and diminished sympathetic responses (Reardon and Malik 1996; Song et al. 2018). In this context, a study by Song et al. (2018) of 2212 elderly nursing home patients showed that deintensification of antihypertensive medications led to less falls, but an overall increase in 30-day mortality.

Our study also revealed that CKD was significantly associated with poorer clinical outcomes. Tran et al. (2019) studied the effects that CKD has on geriatric, community-dwelling individuals and found that decreasing eGFR was independently associated with increasing severity of gait cycle abnormalities. These gait abnormalities manifested as an elevated risk for falls (hazard ratio 1.72) (Tran et al. 2019). This elevated risk was independent of being a geriatric patient (Ziere et al. 2006), as well as other manifestations of CKD including renal osteodystrophy (Goto et al. 2020). In our study, we found that nearly 70% of patients in our overall population experienced a gait-related fall (same level/stairs), which was consistent with those patients with CKD. Our analyses expand on these results and illustrate that patients with CKD/ESRD are at significantly elevated risk for poorer clinical courses after trauma-related falls in nearly all outcomes studied, including ICU admission, increased length of hospital stay, and 30-day mortality. Other studies have also demonstrated the poorer prognosis of patients with renal insufficiency after a fall by identifying an increased association for fracture as well as risk attributed to hemodialysis (Goto et al. 2020; Wang et al. 2020; Tinetti et al. 1988; Rossier et al. 2012). Our findings augment the serious risks that geriatric patients with renal abnormalities face as a result of falls. Strikingly, our analyses reveal that patients with a prior history of CKD/ESRD have over a 2.5 × risk of 30-day mortality compared to those without when presenting to the emergency department after a fall. These results have implications that question whether more fall-related precautions and functional status evaluations should be undertaken for elderly patients with CKD/ESRD to avoid these consequences. Additionally, it suggests that geriatric patients with significant renal disease should be evaluated after a fall with a higher clinical index of suspicion of sequelae to mitigate poorer clinical outcomes. A potential limitation to our study is that our analysis did not stratify based on the stage of CKD, the severity of ESRD, or whether maintenance hemodialysis was required. Further studies investigating whether the risks of these poorer outcomes are graded based on the severity of renal insufficiency could contribute more to evaluating the significant risk of poorer clinical outcomes in this population.

In addition to renal insufficiency producing significantly poorer hospital outcomes, our data also revealed that a history of malignancy, current or past, is an independent predictor of 30-day mortality. Spoelstra et al. (2013) found that an increased risk in falls in those with malignancy is 1.16 (95% CI 1.01–1.33) compared to those without cancer. We theorize that frailty associated with malignancy could be a contributing factor to the increased fall risk. Frailty is prevalent in those who have survived or currently have cancer and characterized by diminished physiologic reserve that negatively impact function and longevity (Guida et al. 2019). A review article from Ness and Wogksch (2020) noted that rates of frailty are common in older individuals, at around 11%, while cancer survivors have a median frailty rate of 42–43%. Several studies (Fried et al. 2001; Ensrud et al. 2007) have demonstrated frailty to be a significant risk factor for falls in the geriatric population. Our results elaborate on these findings and conclude that history of malignancy, leading to frailty, is a statistically significant predictor of 30-day mortality after fall leading to an emergency department visit. It has also been postulated that chemotherapy-induced peripheral neuropathy plays a role in the increased fall risk in the cancer population and that the associated burden of malignancy may lead to a poorer overall prognosis. However, studies have found conflicting evidence regarding the association between CIPN and falls (Komatsu et al. 2019; Gewandter et al. 2013). Regardless, our analyses suggest the clinical importance of considering malignancy history when approaching trauma-related falls in the geriatric population. Treatment and goals of care discussions should be informed by malignancy history to affirm clinical decisions and reduce poorer outcome risk. We postulate that these risks may be modified if stratified for active malignancy versus chronologic history of malignancy and emphasize this as a point of future research.

### Strengths and limitations

The strengths of our study include a large population of geriatric patients at Level 1 Trauma Center in New York City. Our population was drawn from an ethnically diverse community with a wide array of comorbidities allowing for a wide scope of analyses.

However, our study may have been limited by the retrospective methodology in that although numerous significant associations were described, causation cannot be assumed. This study also has all limitations inherent to a single-institution retrospective clinical study. Additionally, due to the large quantity of data being gathered from electronic medical records, information bias and reporting bias secondary to inadequate charting or incomplete medication lists could have affected results. This is particularly relevant for our analyses regarding ethnicity and race as they were self-reported in the EMR and many times unable to be fully distinguished. This was particularly true for patients identifying as Hispanic ethnicity, as we did not have information regarding their race (e.g., black, white, etc.).

Our hospital EMR included all hospital encounters for each patient, thus we believe that we were able to identify most of the medications prescribed and comorbidities. However, incomplete charting or lack of patient reporting could have affected these data points. We also queried the EMR to identify those who required readmission to the hospital for any health-related event within 30 days. This limited our ability to accurately count all patients that required hospital readmission as those that followed up at alternate hospitals would not have been captured. This limitation could have also affected outcomes regarding mortality, as patients that expired at home or at another institution would not have been identified.

Due to the amount of data points collected and the large number of statistical analyses performed for this study, there was an increased chance for positive associations and Type 1 error. For example, certain positive associations may have been attributed to a small number of patients experiencing a particular outcome (e.g., Asian race associated with ICU admission, but decreased mortality; heparin use associated with decreased hospital admission, but increased length of stay if admitted). Additionally, we did not collect information on all classes of medications prescribed (e.g., diabetic medications, statins, type of antihypertensive agents), as well as medication adherence. A more robust analysis in this area could have strengthened our paper.

## Conclusion

Fall-related emergency department visits are common in the older adult population, and poor clinical outcomes of these visits may be prevented by a better understanding of predictive risk factors for these outcomes. We found that polypharmacy was associated with an increased risk of hospitalization, in terms of both primary admission and readmission rates following a fall. Although patients with numerous comorbidities may benefit from additional medication to support their functional status, providers should be vigilant that increased medication burden may affect clinical outcomes after falls. We also found that renal disease and malignancy history may be contributing factors to poorer outcomes after a fall in the geriatric population. Further research in this area is needed to fully address the connection between these factors and poor clinical outcomes. Understanding comorbidities and medication burden is an important facet in triaging geriatric fall-related emergency department visits.

### Supplementary Information

**Additional file 1: Table S1.** Hospital admission (unadjusted analysis). **Table S2**. Length of stay (unadjusted analysis). **Table S3.** Intensive care unit admission (unadjusted analysis). **Table S4.** 30-day mortality (unadjusted analysis). **Table S5.** Readmission (unadjusted analysis).

## Data Availability

The datasets used and/or analyzed during the current study are available from the corresponding author upon reasonable request.

## Abbreviations

BMI: Body mass index
ICU: Intensive care unit
NOAC: Novel oral anticoagulants
ASA: Aspirin
CHF: Congestive heart failure
CAD: Coronary artery disease
COPD: Chronic obstructive pulmonary disease
CKD/ESRD: Chronic kidney disease/end-stage renal disease
CVA: Cerebrovascular accident
OR: Odds ratio
aOR: Adjusted odds ratio
MOF: Mechanism of fall
