# Supplementary material for: Trauma-related falls in an urban geriatric population: predictive risk factors for poorer clinical outcomes
Source: Inj Epidemiol. 2023 Jan 30;10:7. doi: 10.1186/s40621-023-00418-9 (PMC9887835; doi:10.1186/s40621-023-00418-9)
Supplement: Supplementary file 1 — Additional file 1: Table S1. Hospital admission (unadjusted analysis). Table S2. Length of stay (unadjusted analysis). Table S3. Intensive care unit admission (unadjusted analysis). Table S4. 30-day mortality (unadjusted analysis). Table S5. Readmission (unadjusted analysis). [file 40621_2023_418_MOESM1_ESM.docx]

| **Supplementary Table 1 Hospital Admission. (Unadjusted Analysis)** |  |  |  |  |
| --- | --- | --- | --- | --- |
| **Comorbidity/Risk Factor** | **Presence of Comorbidity** | | **Significance** | **Odds Ratio with 95% CI** |
|  | YES | NO |  |  |
| **Factor** |  |  |  |  |
| *Age* |  |  | 0.387 |  |
| *Race* |  |  | 0.607 |  |
| *BMI* |  |  | 0.09 |  |
| *Male* | 78.9% | 82.1% | 0.19 | 0.82 (0.60 - 1.10) |
| *Polypharmacy ≥ 4* | 82.6% | 71.1% | <.001 | 1.92 (1.33 - 2.78) |
| *Polypharmacy ≥ 10* | 84.4% | 78.7% | 0.026 | 1.46 (1.04 - 2.04) |
| **Prescribed Medications** |  |  |  |  |
| *Anticoagulation* | 85.1% | 78.3% | 0.008 | 1.58(1.13 - 2.21) |
| *Aspirin* | 78.3% | 82.5% | 0.079 | 0.76 (0.56 - 1.03) |
| *NOAC* | 83.6% | 80.0% | 0.236 | 1.28 (0.85 - 1.91) |
| *Heparin* | 61.1% | 81.0% | 0.034 | 0.37 (0.14 - 0.96) |
| *Warfarin* | 76.7% | 80.8% | 0.508 | 0.78 (0.38 - 1.61) |
| *Antiplatelet (not ASA)* | 80.6% | 80.7% | 0.980 | 0.99 (0.63 - 1.56) |
| *Antihypertensive* | 78.5% | 85.0% | 0.013 | 0.64 (0.46 - 0.91) |
| *Diuretic* | 83.3% | 79.4% | 0.150 | 1.29 (0.91 - 1.82) |
| *Antidepressant* | 82.2% | 80.3% | 0.559 | 1.13 (0.74 - 1.71) |
| *Narcotic* | 78.9% | 80.8% | 0.696 | 0.89 (0.503 - 1.583) |
| **Comorbid Condition** |  |  |  |  |
| *Hypertension* | 80.3% | 82.2% | 0.546 | 0.88 (0.59 - 1.33) |
| *Depression* | 83.2% | 80.0% | 0.317 | 1.24 (0.81 - 1.89) |
| *Diabetes* | 80% | 80.9% | 0.72 | 0.95 (0.68 - 1.29) |
| *Hyperlipidemia* | 84.4% | 76.1% | <.001 | 1.69 (1.24 - 2.29) |
| *Atrial Fibrillation* | 80.6% | 80.6% | 0.98 | 0.99 (0.68 - 1.45) |
| *Congestive Heart Failure* | 82.1% | 80.4% | 0.62 | 1.12 (0.70 - 1.78) |
| *Coronary Artery Disease* | 81.8% | 80.1% | 0.521 | 1.12 (0.79 - 1.57) |
| *History of CVA* | 77.7% | 81.0% | 0.369 | 0.81 (0.52 - 1.27) |
| *History of Malignancy* | 84.3% | 80.0% | 0.209 | 1.35 (0.84 - 2.14) |
| *CKD/ESRD* | 92.8% | 78.4% | <.001 | 3.55 (1.93 - 6.52) |
| *COPD* | 82.2% | 80.4% | 0.608 | 1.12 (0.72 - 1.74) |
| *History of Dementia* | 78.0% | 81.6% | 0.188 | 0.80 (0.56 - 1.12) |

| **Supplementary Table 2.**  **Length of Stay (Days) (Unadjusted Analysis)** |  |  |  |
| --- | --- | --- | --- |
| **Comorbidity/Risk Factor** | **LOS with and without Comorbidity with 95% CI** | | **Significance** |
|  | YES | NO |  |
| **Factor** |  |  |  |
| *Age* |  |  | 0.81 |
| *Race* |  |  | 0.424 |
| *BMI* |  |  | 0.496 |
| *Male* | 5.32 (4.58 - 6.06) | 4.35 (3.97 - 4.72) | 0.012 |
| *Polypharmacy ≥ 4* | 4.82 (4.40 - 5.15) | 4.56 (3.68 - 5.45) | 0.639 |
| *Polypharmacy ≥ 10* | 5.02 (4.29 - 5.74) | 4.65 (4.22 - 5.08) | 0.361 |
| **Prescribed Medications** |  |  |  |
| *Anticoagulation* | 4.77 (4.40 - 5.16) | 4.79 (4.33 - 5.25) | 0.962 |
| *Aspirin* | 3.96 (3.44 - 4.48) | 5.42 (4.89 - 5.95) | <.001 |
| *NOAC* | 4.86 (3.97 - 5.75) | 4.76 (4.34 - 5.17) | 0.826 |
| *Heparin* | 8.38 (2.12 - 14.65) | 4.73 (4.35 - 5.10) | 0.028 |
| *Warfarin* | 6.73 (4.26 - 9.20) | 4.70 (4.32 - 5.08) | 0.042 |
| *Antiplatelet (not ASA)* | 4.34 (3.05 - 5.62) | 4.84 (4.46 - 5.23) | 0.369 |
| *Antihypertensive* | 4.86 (4.41 - 5.31) | 4.60 (3.91 - 5.29) | 0.532 |
| *Diuretic* | 4.75 (4.12 - 5.39) | 4.79 (4.32 - 5.26) | 0.932 |
| *Antidepressant* | 4.33 (3.35 - 5.30) | 4.87 (4.46 - 5.28) | 0.282 |
| *Narcotic* | 5.64 (4.06 - 7.21) | 4.71 (4.32 - 5.10) | 0.225 |
| **Comorbid Condition** |  |  |  |
| *Hypertension* | 4.77 (4.37 - 5.17) | 4.77 (3.79 - 5.83) | 0.998 |
| *Depression* | 4.29 (3.33 - 5.25) | 4.88 (4.47 - 5.29) | 0.23 |
| *Diabetes* | 4.68 (4.10 - 5.26) | 4.82 (4.34 - 5.31) | 0.72 |
| *Hyperlipidemia* | 4.33 (3.89 - 4.77) | 5.38 (4.72 - 6.04) | 0.007 |
| *Atrial Fibrillation* | 5.47 (4.51 - 6.44) | 4.59 (4.19 - 4.99) | 0.064 |
| *Congestive Heart Failure* | 5.58 (4.44- 6.73) | 4.65 (4.25 - 5.05) | 0.098 |
| *Coronary Artery Disease* | 5.07 (4.22 - 5.91) | 4.65 (4.25 - 5.05) | 0.315 |
| *History of CVA* | 5.26 (3.78 - 6.74) | 4.71 (4.33 - 5.09) | 0.366 |
| *History of Malignancy* | 5.17 (4.17 - 6.18) | 4.70 (4.30 - 5.11) | 0.383 |
| *CKD/ESRD* | 6.84 (5.74 - 7.94) | 4.36 (3.97 - 4.75) | <.001 |
| *COPD* | 6.21 (4.76 - 7.65) | 4.52 (4.16 - 4.88) | 0.002 |
| *History of Dementia* | 4.74 (4.01 - 5.48) | 4.78 (4.34 - 5.22) | 0.933 |

| **Supplementary Table 3.**  **ICU Admission. (Unadjusted Analysis)** |  |  |  |  |
| --- | --- | --- | --- | --- |
| **Comorbidity/Risk Factor** | **Presence of Comorbidity** | | **Significance** | **Odds Ratio with 95% CI** |
|  | YES | NO |  |  |
| **Factor** |  |  |  |  |
| *Age* |  |  |  | 0.122 |
| *Race* |  |  |  | 0.025 |
| *BMI* |  |  |  | 0.756 |
| *Male* | 16.3% | 11.4% | 0.021 | 1.51 (1.06 - 2.15) |
| *Polypharmacy ≥ 4* | 14.2% | 9.9% | 0.127 | 1.49 (0.89 - 2.52) |
| *Polypharmacy ≥ 10* | 12.6% | 13.9% | 0.566 | 0.89 (0.62 - 1.30) |
| **Prescribed Medications** |  |  |  |  |
| *Anticoagulation* | 14.4% | 13.1% | 0.574 | 1.11 (0.77 - 1.59) |
| *Aspirin* | 12.4% | 14.3% | 0.350 | 0.84 (0.59 - 1.20) |
| *NOAC* | 14.5% | 13.2% | 0.631 | 1.11 (0.72 - 1.71) |
| *Heparin* | 16.7% | 13.4% | 0.689 | 1.29 (0.37 - 4.51) |
| *Warfarin* | 25.6% | 13.0% | 0.018 | 2.30 (1.13 - 4.68) |
| *Antiplatelet (not ASA)* | 13.0% | 13.5% | 0.875 | 0.96 (0.56 - 1.62) |
| *Antihypertensive* | 13.2% | 14.0% | 0.717 | 0.93 (0.64 - 1.36) |
| *Diuretic* | 15.2% | 12.8% | 0.301 | 1.22 (0.83 - 1.78) |
| *Antidepressant* | 12.8% | 13.6% | 0.765 | 0.93 (0.58 - 1.50) |
| *Narcotic* | 7.9% | 13.9% | 0.140 | 0.53 (0.23 - 1.25) |
| **Comorbid Condition** |  |  |  |  |
| *Hypertension* | 13.9% | 11.5% | 0.374 | 1.25 (0.77 - 2.02) |
| *Depression* | 11.8% | 13.9% | 0.453 | 0.83 (0.51 - 1.34) |
| *Diabetes* | 13.2% | 13.7% | 0.837 | 0.96 (0.67 - 1.39) |
| *Hyperlipidemia* | 13.9% | 13.1% | 0.675 | 1.08(0.76 - 1.53) |
| *Atrial Fibrillation* | 18.5% | 12.3% | 0.016 | 1.63 (1.09 - 2.42) |
| *Congestive Heart Failure* | 14.3% | 13.4% | 0.782 | 1.08 (0.66 - 1.79) |
| *Coronary Artery Disease* | 15.3% | 12.8% | 0.296 | 1.22 (0.83 - 1.78) |
| *History of CVA* | 19.4% | 12.7% | 0.038 | 1.65 (1.02 - 2.65) |
| *History of Malignancy* | 16.3% | 13.1% | 0.274 | 1.29 (0.81 - 2.08) |
| *CKD/ESRD* | 19.9% | 12.4% | 0.01 | 1.76 (1.14 - 2.70) |
| *COPD* | 14.0% | 13.5% | 0.855 | 1.05 (0.64 - 1.71) |
| *History of Dementia* | 12.5% | 13.9% | 0.562 | 0.89 (0.59 - 1.33) |

| **Supplementary Table 4.**  **30-Day Mortality (Unadjusted Analysis)** |  |  |  |  |
| --- | --- | --- | --- | --- |
| **Comorbidity/Risk Factor** | **Presence of Comorbidity** | | **Significance** | **Odds Ratio with 95% CI** |
|  | YES | NO |  |  |
| **Factor** |  |  |  |  |
| *Age* |  |  |  | 0.168 |
| *Race* |  |  |  | 0.964 |
| *BMI* |  |  |  | 0.235 |
| *Male* | 3.2% | 2.0% | 0.230 | 1.59 (0.74 - 3.41) |
| *Polypharmacy ≥ 4* | 2.2% | 3.9% | 0.204 | 0.57 (0.24 - 1.37) |
| *Polypharmacy ≥ 10* | 1.6% | 3.0% | 0.186 | 0.54 (0.22 - 1.36) |
| **Prescribed Medications** |  |  |  |  |
| *Anticoagulation* | 1.6% | 3.0% | 0.178 | 0.54 (0.22 - 1.34) |
| *Aspirin* | 2.1% | 2.8% | 0.476 | 0.75 (0.34 - 1.66) |
| *NOAC* | 1.4% | 2.8% | 0.274 | 0.52 (0.15 - 1.73) |
| *Heparin* | 5.6% | 2.5% | 0.407 | 2.33 (0.29 - 18.13) |
| *Warfarin* | 4.7% | 2.4% | 0.362 | 1.96 (0.45 - 8.55) |
| *Antiplatelet (not ASA)* | 2.9% | 2.5% | 0.772 | 1.17 (0.40 - 3.44) |
| *Antihypertensive* | 2.4% | 2.7% | 0.813 | 0.91 (0.40 - 2.04) |
| *Diuretic* | 3.2% | 2.2% | 0.352 | 1.45 (0.66 - 3.21) |
| *Antidepressant* | 3.9% | 2.2% | 0.204 | 1.75 (0.73 - 4.21) |
| *Narcotic* | 5.3% | 2.3% | 0.113 | 2.35 (0.79 - 6.98) |
| **Comorbid Condition** |  |  |  |  |
| *Hypertension* | 2.5% | 2.6% | 0.919 | 0.95 (0.35 - 2.54) |
| *Depression* | 3.8% | 2.3% | 0.231 | 1.70 (0.71 - 4.07) |
| *Diabetes* | 2.4% | 2.6% | 0.893 | 0.94 (0.42 - 2.12) |
| *Hyperlipidemia* | 2.7% | 2.2% | 0.599 | 1.23 (0.57 - 2.68) |
| *Atrial Fibrillation* | 2.8% | 2.4% | 0.772 | 1.15 (0.46 - 2.87) |
| *Congestive Heart Failure* | 3.6% | 2.4% | 0.391 | 1.53 (0.57 - 4.12) |
| *Coronary Artery Disease* | 4.2% | 1.8% | 0.023 | 2.37 (1.09 - 5.09) |
| *History of CVA* | 2.3% | 2.5% | 0.884 | 0.91 (0.27 - 3.08) |
| *History of Malignancy* | 6.5% | 1.8% | <.001 | 3.72 (1.67 - 8.28) |
| *CKD/ESRD* | 5.4% | 2.0% | 0.01 | 2.81 (1.24 - 6.36) |
| *COPD* | 3.2% | 2.4% | 0.563 | 1.33(0.50 - 3.58) |
| *History of Dementia* | 2.4% | 2.6% | 0.856 | 0.92 (0.39 - 2.21) |

| **Supplementary Table 5.**  **30-Day Readmission (Unadjusted Analysis)** | |  |  |  |
| --- | --- | --- | --- | --- |
| **Comorbidity/Risk Factor** | **Presence of Comorbidity** | | **Significance** | **Odds Ratio with 95% CI** |
|  | YES | NO |  |  |
| **Factor** |  |  |  |  |
| *Age* |  |  |  | 0.005 |
| *Race* |  |  |  | 0.679 |
| *BMI* |  |  |  | 0.308 |
| *Male* | 15.8% | 10.0% | 0.005 | 1.67 (1.16 - 2.42) |
| *Polypharmacy ≥ 4* | 13.70% | 6.10% | 0.005 | 2.45 (1.29 - 4.65) |
| *Polypharmacy ≥ 10* | 16.70% | 10.20% | 0.002 | 1.76 (1.22 -2.54) |
| **Prescribed Medications** |  |  |  |  |
| *Anticoagulation* | 11.9% | 13.2% | 0.525 | 1.13 (0.78 - 1.65) |
| *Aspirin* | 14.7% | 10.6% | 0.043 | 1.45 (1.01 - 2.09) |
| *NOAC* | 11.1% | 12.7% | 0.529 | 0.86 (0.53 - 1.38) |
| *Heparin* | 16.7% | 12.3% | 0.580 | 1.42 (0.41 - 4.98) |
| *Warfarin* | 9.3% | 12.5% | 0.529 | 0.72 (0.25 - 2.04) |
| *Antiplatelet (not ASA)* | 16.5% | 11.8% | 0.112 | 1.48 (0.91 - 2.42) |
| *Antihypertensive* | 11.1% | 15.2% | 0.059 | 0.70 (0.48 - 1.02) |
| *Diuretic* | 14.5% | 11.6% | 0.19 | 1.29 (0.88 - 1.90) |
| *Antidepressant* | 14.9% | 11.9% | 0.261 | 1.30 (0.82 - 2.05) |
| *Narcotic* | 13.2% | 12.3% | 0.837 | 1.06 (0.54 - 2.15) |
| **Comorbid Condition** |  |  |  |  |
| *Hypertension* | 12.1% | 13.6% | 0.570 | 0.88 (0.55 - 1.39) |
| *Depression* | 16.7% | 11.5% | 0.051 | 1.54 (0.99 - 2.39) |
| *Diabetes* | 14.3% | 11.4% | 0.169 | 1.30 (0.89 - 1.88) |
| *Hyperlipidemia* | 15.4% | 8.8% | <.001 | 1.90 (1.29 - 2.80) |
| *Atrial Fibrillation* | 10.2% | 12.9% | 0.284 | 0.77 (0.47 - 1.25) |
| *Congestive Heart Failure* | 18.6% | 11.5% | 0.017 | 1.76 (1.10 - 2.82) |
| *Coronary Artery Disease* | 13.0% | 12.1% | 0.703 | 1.08 (0.72 - 1.60) |
| *History of CVA* | 10.1% | 12.7% | 0.397 | 0.77 (0.42 - 1.41) |
| *History of Malignancy* | 9.2% | 12.9% | 0.190 | 0.68 (0.38 - 1.22) |
| *CKD/ESRD* | 18.0% | 11.4% | 0.017 | 1.71 (1.09 - 2.66) |
| *COPD* | 16.6% | 11.7% | 0.086 | 1.50 (0.94 - 2.39) |
| *History of Dementia* | 11.5% | 12.7% | 0.599 | 0.89 (0.59 - 1.36) |

HIGHLIGHT = Statistically significant on univariate analysis and included in multivariate regression
